# Implementing mobile eye tracking in psychological research: A practical guide

**DOI:** 10.3758/s13428-024-02473-6

**Published:** 2024-08-15

**Authors:** Xiaoxue Fu, John M. Franchak, Leigha A. MacNeill, Kelley E. Gunther, Jeremy I. Borjon, Julia Yurkovic-Harding, Samuel Harding, Jessica Bradshaw, Koraly E. Pérez-Edgar

**Affiliations:** 1 Department of Psychology, University of South Carolina, Columbia, SC USA; 2 Department of Psychology, University of California Riverside, Riverside, CA USA; 3 Department of Medical Social Sciences, Northwestern University, Feinberg School of Medicine, Northwestern University, Chicago, IL USA; 4 Institute for Innovations in Developmental Sciences, Northwestern University, Evanston, IL USA; 5 Neuroscience and Cognitive Science Program, University of Maryland, College Park, MD USA; 6 Department of Psychology, University of Houston, Houston, TX USA; 7 Texas Institute for Measurement, Evaluation, and Statistics, University of Houston, Houston, TX USA; 8 Texas Center for Learning Disorders, University of Houston, Houston, TX USA; 9 Department of Psychology, The Pennsylvania State University, University Park, PA USA

**Keywords:** Mobile eye tracking, Head-mounted eye tracking, Method, Technology

## Abstract

Eye tracking provides direct, temporally and spatially sensitive measures of eye gaze. It can capture visual attention patterns from infancy through adulthood. However, commonly used screen-based eye tracking (SET) paradigms are limited in their depiction of how individuals process information as they interact with the environment in “real life”. Mobile eye tracking (MET) records participant-perspective gaze in the context of active behavior. Recent technological developments in MET hardware enable researchers to capture egocentric vision as early as infancy and across the lifespan. However, challenges remain in MET data collection, processing, and analysis. The present paper aims to provide an introduction and practical guide to starting researchers in the field to facilitate the use of MET in psychological research with a wide range of age groups. First, we provide a general introduction to MET. Next, we briefly review MET studies in adults and children that provide new insights into attention and its roles in cognitive and socioemotional functioning. We then discuss technical issues relating to MET data collection and provide guidelines for data quality inspection, gaze annotations, data visualization, and statistical analyses. Lastly, we conclude by discussing the future directions of MET implementation. Open-source programs for MET data quality inspection, data visualization, and analysis are shared publicly.

## Introduction

Eye movements provide a window into one’s perception, cognition, and visually guided behavior. Eye movements can indicate the deployment of visual attention (Henderson, 2003). Attention, in turn, acts as a processing mechanism that filters out excessive information from the environment by biasing selection based on the individual’s current goals (Desimone & Duncan, 1995) and affective states (Todd et al., 2012). Visual experiences influence downstream cognition, learning, action, and affect (Crick & Dodge, 1994). Controlled laboratory experiments often study visual attention in isolation. However, in everyday life, visual attention is closely linked to the individual’s ongoing behavior and experiences of the physical and social environments (Franchak, 2020a; Hayhoe & Rothkopf, 2011). While we have gained tremendous insights from screen-based tasks, without studying attention in situ, we can only approximate how attention, action, and social information dynamically influence each other in real-time and in real-life environments.

Head-mounted, or mobile eye tracking (MET), records eye movements embedded in an individual’s free-flowing behaviors as they interact with the environment. The technology has been pioneered in adults since the early 1900s (Land, 2006). MET systems have become more portable and robust with technological advancement. This recent development facilitates research into attention during active visual exploration (Ballard et al., 1997), especially in infants and young children (Franchak, 2017; Franchak, 2019). MET typically consists of a scene camera that captures the wearer’s first-person view and one or two eye cameras that support monocular or binocular eye tracking, respectively. The MET system records the wearer’s gaze direction and maps the three-dimensional gaze point to the two-dimensional space of the scene camera, allowing researchers to visualize the point of gaze overlaid on the scene camera recording (Macinnes et al., 2018).

The use of MET yields several key advantages. Compared to video recordings, MET provides a more proximal, temporally and spatially sensitive measure of attention from the first-person perspective (Franchak, 2019; Franchak, 2020b; Fu & Pérez-Edgar, 2019; Pérez-Edgar et al., 2020). MET captures rich micro-longitudinal data by sampling looking locations within self-generated behavior for extended periods of time (see Data visualization section). MET data can then be used to probe within-person changes of attention over time and capture the moment-to-moment dynamics between the environmental inputs, individuals’ attention, and behavior (see Data analysis section). Hence, MET studies may provide new understandings of human cognition operating within the individual’s active motor and social behaviors (Ballard et al., 1997; Gibson, 1979; Yoshida & Burling, 2011).

The present paper provides an introduction and practical guide for MET data collection, processing, and analytic methods to new researchers in the field. Existing literature has highlighted the utility and advantages of MET (Franchak, 2019, 2020a; Pérez-Edgar et al., 2020; Yoshida & Burling, 2011), technical challenges (Hessels, Niehorster et al., 2020b; Niehorster et al., 2020; Valtakari et al., 2021), and provided practical guides in MET data collection and data quality inspection (Franchak & Yu, 2022; Hooge et al., 2023; Niehorster et al., 2023; Slone et al., 2018). The present paper complements and extends existing method papers by providing a review of current MET methodologies and practical guidance that are applicable to MET research that covers a wide age span from infancy to adulthood. We will briefly review studies that illustrate the utility of MET as an integral tool for understanding attentional processes in locomotion, learning, and social interactions in adults, children, and infants in the "The utility of MET technology" section. This is followed by recommendations on MET data collection in the "MET data collection considerations" section, data quality assessment in the "MET data quality inspection" section, gaze annotation methods in the "Gaze annotations" section, visualization of looking events in the "Data visualization" section, and data analysis approaches in the "Data analysis" section. Methods introduced in these sections are applicable to MET research with adults, children, and infants. Finally, we will discuss remaining challenges and future directions in the "Future directions" section. In addition to reviewing existing tools, the present paper also provides computer programs and example data for demonstrating methods for data quality assessment, data visualization, and data analysis (https://github.com/xiaoxuefu/MET_methods). The example MET data were collected from two research projects: the iTRAC study that enrolled 5- to 7-year-olds and the ACTION study that involves infants at 4 and 8 months of age. Descriptions of the two projects are provided in the GitHub repository. Table 1 also lists information on open-access MET data and data analytic tools provided by studies cited in the present paper.

**Table 1:** Open-access data and tools cited

Name	Authors	URL
Mobile eye tracking data		
Gaze-in-Wild	Franchak et al. (2018)	https://nyu.databrary.org/volume/135
	Kothari et al. (2020)	https://www.cis.rit.edu/~rsk3900/gaze-in-wild/
	Matthis et al. (2018)	10.6084/m9.figshare.6130850.
Glasses Test	Niehorster et al. (2020)	https://github.com/dcnieho/GlassesTestCodeData
Data Quality Assessment		
GlassesValidator	Niehorster et al. (2023)	https://github.com/dcnieho/glassesValidator
Gaze Annotations		
GazeCode	Benjamins et al. (2018)	https://github.com/jsbenjamins/gazecode
ROI Coder	Franchak (unpublished)	https://github.com/JohnFranchak/roi_coder
Eye Tracker Analysis	Jongerius et al. (2021)	https://osf.io/4uy35/?view_only=785a011774cf4c4f8c5e4608b34a2a38

## The utility of MET technology

### MET as a tool to examine cognition embodied in individuals’ sensorimotor systems

MET research has provided empirical evidence for embodied cognition (Ballard et al., 1997; Yoshida & Burling, 2011). The ecological approach suggests that visual attention operates in conjunction with a whole-body locomotor system (Gibson, 1979). Research has historically studied attention and active locomotor behavior as two separate, encapsulated systems. MET opens the opportunity for examining the “what” and “when” of visual attention during sequences of actions carried out during everyday activities (Hayhoe, 2017, 2018; Hayhoe & Rothkopf, 2011) or other fieldworks (e.g., fly a plane: Socha et al., 2022; perform a clinical procedure: Wright et al., 2022). MET research in adults reveals the tight spatial and temporal coupling between attention, action, and task demands (Hayhoe et al., 2003; Land et al., 1999). For example, when adults walk on complex terrain, they gaze at the point at which they would place their foot two steps ahead (Domínguez-Zamora & Marigold, 2019; Marigold & Patla, 2007; Matthis & Fajen, 2014), and adjust the timing of fixations to match the difficulty of foot placement. When walking over flat terrain, adults can navigate obstacles without the need to fixate (Franchak & Adolph, 2010). When adults navigate crowds, participants avoid eye contact as instructed by orienting both their heads and eyes towards the floor (Hessels et al., 2022). Hence, the synergistic eye-body coordination is constantly adjusted in real time based on in-the-moment behavioral goals.

MET recordings in infants reveal that the development of gross motor (e.g., posture) and fine motor (e.g., manual object manipulation) skills shape infant attentional behavior. For example, crawling infants (13-month-olds) look mostly at the floor, whereas age-matched infants who walk can see more distal objects and people (Kretch et al., 2014; Luo & Franchak, 2020). Moreover, infants (12-month-olds) look more at their caregivers’ faces when upright and sitting, compared to when in a prone position (Franchak et al., 2018). As infants’ fine motor skills mature, manual object explorations generate more salient and variable object images in the visual field (15–25 months). Visual inputs in the real world are maintained in infants (12–24 months) by consistently aligning the head and eyes while looking at objects (Borjon et al., 2021). The visual inputs facilitate learning of word-object associations (Bambach et al., 2018; Slone et al., 2019; Yu & Smith, 2012). These studies collectively underscore the importance of studying visual attention within the developing sensorimotor system.

### MET as a tool to capture social attention embedded in naturalistic interactions

The “second-person” or “person-centered” perspective emphasizes that social attention needs to be examined in the context of the individuals’ interaction with social partners (Fu & Pérez-Edgar, 2019; Pérez-Edgar et al., 2020; Redcay & Schilbach, 2019; Risko et al., 2012). In real-life social interactions, eye gaze serves the dual function of both collecting and communicating information (Gobel et al., 2015; Nasiopoulos et al., 2015). By recording visual attention during real-time social behaviors, MET is a unique tool for understanding the dual function of eye gaze. For example, MET studies found that adults tend to avoid directly looking at strangers when engaging in first-person social interactions, compared to being passive observers (Foulsham et al., 2011; Freeth et al., 2013; Laidlaw et al., 2011). The behavior might be driven by the implicit understanding of social customs and the effect of gaze in delivering social information. However, there are cultural differences in social attention, such that East Asians engaged in more mutual gaze than Western Caucasians during face-to-face conversations (Haensel et al., 2022). Adults also utilize eye gaze as social communicative cues. For example, when verbal instructions for a task activity are ambiguous, participants are more likely to follow the gaze of their social partners compared to when given unambiguous verbal instructions (Macdonald & Tatler, 2013). Hence, social attention is context-driven and goal-directed.

MET is also an indispensable tool for understanding the coupling between attention and affective behavior. Vallorani et al. (2022) showed that among 5- to 7-year-olds, a child’s expression of positive affect predicts a greater likelihood of looking at peers during dyadic free play. Social attention, in turn, is linked to a greater likelihood of the child expressing positive affect when the peer is expressing neutral affect. Existing MET studies in adults and children underscore the importance of studying social attention nested in the individuals’ affect and social experiences (Fu & Pérez-Edgar, 2019; Pérez-Edgar et al., 2020). One application of measuring social attention embedded in real-life interactions is to study threat-related attention bias linked to risk for internalizing symptoms (Fu & Pérez-Edgar, 2019). Behavioral inhibition, a temperament profile characterized by heightened vigilance and reactivity to novelty in infancy and social reticence in childhood, is a robust risk factor for anxiety disorders (Chronis-Tuscano et al., 2009; Clauss & Blackford, 2012). During a relatively benign social encounter, children (partially overlapping sample as Vallaroni et al., 2022) with high behavioral inhibition show greater attention avoidance towards an adult stranger (Fu et al., 2019). Moreover, children with an attention profile characterized by avoidance to an adult stranger exhibit greater internalizing symptoms even when controlling for their behavioral inhibition level (Gunther et al., 2022). When encountering higher social threat (i.e., an adult wearing a “scary” mask), children with high behavioral inhibition showed more attention toward the stranger (Gunther et al., 2021). Together, these findings highlight the importance of studying threat-related attention in the context of naturalistic interactions, as the nature of threat context can influence attention patterns.

Moreover, developmental scientists have used MET to study how learning emerges from free-flowing interactions in infant–caregiver dyads. Joint attention (JA) is a key conduit for language learning. JA reflects children’s ability to coordinate attention with their social partners, creating a critical context for language acquisition (Suarez-Rivera et al., 2022; Tomasello & Farrar, 1986). Traditional laboratory tasks assess infants’ ability to achieve JA by focusing on visual attention patterns, encompassing face looking, gaze following, and object looking (Brooks & Meltzoff, 2005; Tomasello & Farrar, 1986). In contrast, through studying infant–parent free-flowing play behaviors, MET studies in infants and toddlers (9–48 months) show that it is the hand-eye coordination between infants and caregivers, not infants’ visual attention alone, which contributes to the formation of JA (Abney et al., 2020; Yu & Smith, 2013, 2017a, 2017b; Yurkovic-Harding et al., 2022). Parents are more likely to name and touch the toy during bouts of JA, and the multimodal behavior increases infants’ sustained attention to the objects and facilitates real-time learning of the word-referent association (Chen et al., 2021; Suarez-Rivera et al., 2019; Yu & Smith, 2012). As infants actively interact with the environment through sensorimotor (e.g., hand–eye) coordination, they create idiosyncratic inputs for learning (Smith et al., 2018). Hence, MET provides a tool for understanding the formation and characteristics of the environmental inputs from the first-person perspective, and the downstream impacts of these inputs on cognitive development (Yoshida & Burling, 2011).

## MET data collection considerations

Decisions on eye-tracker hardware and MET task procedures are driven by researchers’ requirements regarding (1) participant characteristics, including age, (2) freedom of movement, and (3) data collection environment, such as in controlled laboratory settings or less controlled indoor or outdoor environments (e.g., homes and streets). While the hardware choices and study procedures may vary, a common goal for eye-tracking research is to safeguard data quality, defined as the reliability, validity, and availability of usable data (Hessels & Hooge, 2019; Niehorster et al., 2018). Disruptions of pupil detection (due to factors such as ambient lighting, headset slippage, and eye makeup) and the alignment of the headset relative to the participant’s head (due to movement and slippage) negatively impact data quality (Hessels et al., 2022; Niehorster et al., 2020).

Calibration is a critical procedure for obtaining high data quality. The commonly used video-based pupil-corneal reflection (P-CR) eye tracker records the relative locations of the pupil and corneal reflection. Calibration involves mapping the recorded pupil and corneal reflection locations when the gaze was directed to the calibration targets to the spatial locations of the calibration stimuli (Blignaut et al., 2014). Poor calibration reduces the validity of MET data. Furthermore, care needs to be taken to ensure that experimental manipulations do not create differential impacts on MET data quality between conditions (Hessels et al., 2022). While calibration-free MET devices are commercially available (e.g., Tonsen et al., 2020), we recommend researchers evaluate different calibration options based on participant age and experiment needs. We include information on calibration here, as gaze-estimation accuracy of the calibration-free MET device is yet to be published for children and infants. This section will discuss hardware setup, calibration, and study design issues in example research scenarios based on study considerations on (1) participant characteristics, (2) freedom of movement, and (3) environment. Additional guidance on MET setups is provided in Valtakari et al. (2021) and Slone et al. (2018) for adult and child participants, respectively.

### MET data collection with adults and older children in controlled laboratory environments

Collecting MET data in older participants in controlled environments allows for greater flexibility in hardware setups given the minimal customization required for “out-of-the-box” eye-trackers and participants’ better tolerance and abilities to cooperate (compared to infants and toddlers). One main consideration that determines MET setups is the participants’ freedom of movement. Published studies in participants above 5 years old commonly connect the headset directly to a computer device (e.g., laptop) for data recording and storage (e.g., 5–69 years old: Fu et al., 2019; Hessels et al., 2022; Matthis et al., 2018; Woody et al., 2019). This setup can be burdensome for the participants, and the restrained movement can affect eye–body coordination, a key construct of interest in many MET studies. Newer setups involve connecting the headset to a lightweight smartphone device, which functions as a recording and local storage device (Nasrabadi & Alonso, 2022; Tonsen et al., 2020).

MET research with adults and children who can be instructed to fixate calibration targets has greater flexibility in calibration methods. In a typical calibration session, participants are asked to look at calibration points displayed on a screen, comparable to screen-based eye tracking (SET) calibration (e.g., Fu et al., 2019; Kothari et al., 2020) or a calibration marker fixed on a naturalistic object (e.g., Niehorster et al., 2020; Woody et al., 2019). Studies in this age range may employ online calibration, where the mapping between pupil-corneal-reflection locations and the locations of the calibration points take effect immediately. Offline calibration performs the spatial mapping after data collection. Hence, the advantage of online calibration is allowing for real-time data monitoring and providing the opportunity for just-in-time recalibration.

We recommend a few best practices for performing both online and offline calibration accuracy:

1. **Display calibration targets at a distance comparable to the distance between the participant and primary areas of interest (AOIs). **AOIs refer to the targets of the participants’ looks that will be annotated for data analysis (also see the section “Gaze annotations”). Parallax error is a gaze estimation error introduced when the distance between the wearer and the AOI (i.e., the fixation plane) is different than the distance between the wearer and the calibration target (i.e., the calibration plane). This causes an offset between the true gaze location and the estimated gaze location on the fixation plane and the scene camera coordinate space, which bias the experimenter’s identification of actual gaze location (Mardanbegi & Hansen, 2012; Valtakari et al., 2021). Hence, it is recommended that the calibration targets be presented at the same approximate location of the AOIs. If participants will engage in various viewing distances during the experiment, it is best to perform multiple calibration sessions to accommodate the distance changes or employ a mid-range distance if there is a lack of experimental control on the distance change.
2. **Present multiple calibration targets that cover the participant’s entire field of view (FOV). **We use FOV here to refer to the view of the participants captured by the scene camara. The FOV tends to be smaller than the participant’s visual field, and it is not necessarily equivalent to the reported FOV specifications of a given MET model, depending on factors such as viewing distance, participant’s posture, and the camera angle. Five or more calibration targets can be presented across the participant’s FOV, comparable to SET. This step is to ensure that calibration accuracy is maintained from the center of the FOV to peripheral locations. The experiment should verify that participants do not turn their heads to orient toward peripheral targets, which will result in target clustering in the center of the FOV.
3. **Perform a validation procedure (i.e., calibration check) at the beginning and end of the experiment session, and after any MET headset movement.** A validation procedure is conducted by directing participants to look at specific target locations. Similarly, the targets should be presented in a location that is comparable to the location of the AOIs. Conducting multiple calibration checks during the experiment helps to ensure that the data quality is maintained throughout the experiment. The headset slippage issue can be effectively prevented through monitoring online calibration accuracy and recalibrating to correct accuracy drift (Niehorster et al., 2020). The validation procedure also provides additional calibration points for corrections in offline calibrations. For example, if the eye gaze capture is perturbed by headset movement, the experimenter needs to adjust the eye camera and perform calibration checks. The points of gaze obtained post-adjustment can be used to update the spatial mapping in offline calibration. Finally, performing per-participant validation checks allows reporting of the accuracy metric (see the section “Accuracy”), which can also be used as a control variable in analyses (Franchak & Yu, 2022).

### MET data collection in infants and toddlers

Existing MET studies with infants and toddlers (4–26 months old) in laboratory (e.g., Franchak et al., 2011; Schroer & Yu, 2023; Yu & Smith, 2012, 2017a) and home settings (Bradshaw et al., 2023) have largely followed a common set of equipment setup and calibration procedures. The headset needs to be stably placed on the head to minimize the negative effect of slippage on data quality (Niehorster et al., 2020). Researchers may customize the “out-of-the-box” eye tracker by affixing it on a tailored headband, cap, or beanie for secure placement. For young infants (< 8 months), we recommend utilizing a series of headsets that can accommodate different head sizes, head shapes, and hair textures. Some headsets can be connected to a smartphone to increase children’s mobility (Schroer & Yu, 2023).

It is challenging to instruct infants and toddlers to follow calibration points. Hence, MET studies in this age group commonly implement offline calibration. The calibration procedure can be integrated into a child-experimenter play session during which the experimenter presents engaging calibration targets (e.g., toys and/or laser points) at various locations across the child’s FOV. The calibration target distance from the child and the child’s posture should match the specifications for the formal data collection. Researchers should closely monitor the eye image recording throughout data collection. Additional calibration(s) are required if the eye image capture is perturbed by headset movement. If the study involves interactions with an adult partner, such as a caregiver, the social partner can be trained in calibration target presentations to minimize disruptions during naturalistic interactions. After data collection, a trained researcher marks the calibration target locations on the scene camera recording where the child’s point of gaze is clearly identifiable and directed to the calibration target. An algorithm is then applied to map the pupil and corneal reflection locations with the specified calibration target locations (e.g., Hassoumi et al., 2019). The manual identification of points of gaze and automated mapping procedure are run iteratively to establish satisfactory calibration (Slone et al., 2018).

### MET data collection outside controlled laboratory environments

MET studies have been conducted in naturalistic outdoor (adults: Foulsham et al., 2011; Matthis et al., 2018) and indoor environments (infants at homes: Bradshaw et al., 2023; adults in an event hall: Hessels et al., 2022; a child in a museum: Jung et al., 2018; adults in a clinical setting: Wright et al., 2022). Factors that can then compromise MET data quality include a lack of control over ambient lighting, locations of the target objects (i.e., AOIs), and insufficient calibration procedures (Evans et al., 2012; Hessels et al., 2022). For example, infrared light from the sun when outdoors interferes with pupil and corneal reflection tracking. A remedy is to provide participants with an infrared-blocking visor (Matthis et al., 2018). The distance between the participant and different AOIs can greatly vary. This is both an advantage (greater visual selection) and a disadvantage (greater analytic complexity) of MET. In tasks where participants tilt their heads down, AOIs that are close to the participant and lower than eye level are captured in the lower part of the scene camera view, while farther objects are captured in the higher part of the scene camera view (Slone et al., 2018). Hence, appropriate scene camera positioning needs to be determined to ensure it can capture all AOIs in the study.

Offline calibration can be advantageous in less-controlled environments, given real-time data monitoring might not be possible. Offline calibration offers researchers the opportunity to update the spatial mapping between pupil and corneal reflection locations and the points of gaze directed to the calibration targets after pupil capture is altered by slippage, posture changes, or lighting. The emerging calibration-free MET technology is also promising for maintaining acceptable accuracy in outdoor settings (Tonsen et al., 2020).

## MET data quality inspection

Eye-tracking data quality is quantified by accuracy, precision, and availability of usable data (or data loss) (Hessels & Hooge, 2019). Accuracy is operationalized as the distance (spatial offset) between the gaze location detected by the eye tracker and the actual gaze location measured in degree of visual angle. Precision indexes the level of noise in the eye-tracking data that produces spatial variability between gaze samples. Accuracy and precision provide an index of validity and reliability of the eye-tracking data (Hessels & Hooge, 2019). Data loss can be calculated using the number of valid gaze data points recorded and the expected number of samples based on the specified sampling frequency (Hessels & Hooge, 2019; Hooge et al., 2023; Niehorster et al., 2020).

As illustrated in the "MET data collection considerations" section, there are MET-specific data quality concerns relative to SET studies due to the less constrained nature of MET data collection. After the initial calibration, changes in illumination conditions, the distance between the participant and AOIs, and disruptions in the detection of pupil locations and corneal reflection can introduce errors (Franchak & Yu, 2022; Niehorster et al., 2020). Indeed, the accuracy and precision achieved from the initial calibration may not be maintained at the end of the experiment after unconstrained movement and headset slippage occurred (Niehorster et al., 2020; Santini et al., 2018). Compromised data quality could bias eye-tracking measurements and lead to false conclusions (Wass et al., 2014). Thus, it is critical to examine data quality before data analyses. This section will provide strategies for assessing MET data accuracy, precision, and data loss based on published definitions (Franchak & Yu, 2022; Hessels & Hooge, 2019; Niehorster et al., 2020). We will also discuss strategies to make informed decisions on data analyses based on data quality assessments. An additional pipeline for computing these data quality indices is provided in (Hooge et al., 2023). The authors underscored the importance of inspecting synchronization between the eye and scene camera recordings before computing the data quality indices. While some MET systems provide a built-in function for synchronization, there could still be intermittent periods of asynchronization between the recordings of gaze and target location, which will bias the accuracy index (Hooge et al., 2023).

### Accuracy

One off-the-shelf tool for calculating accuracy is *GlassesValidator* (Niehorster et al., 2023; Table 1). *GlassesValidator* is suited for data collection with adults and older children, as participants are required to look at fixation targets displayed on the poster that is included with the tool. The computation is automated and does not require manual annotations. Briefly, the poster contains arrays of ArUco markers (i.e., barcodes) that allow automated estimation of the participant’s viewing distance and gaze location. A fixation classifier is applied to determine the valid fixation (> 50-ms duration) towards each fixation target. Accuracy is calculated as the deviation between the fixation target and the estimated gaze location in degree of visual angle (i.e., the angle between the line from the eye to the fixation target and the line from the eye to the gaze location).

**Fig. 1:**
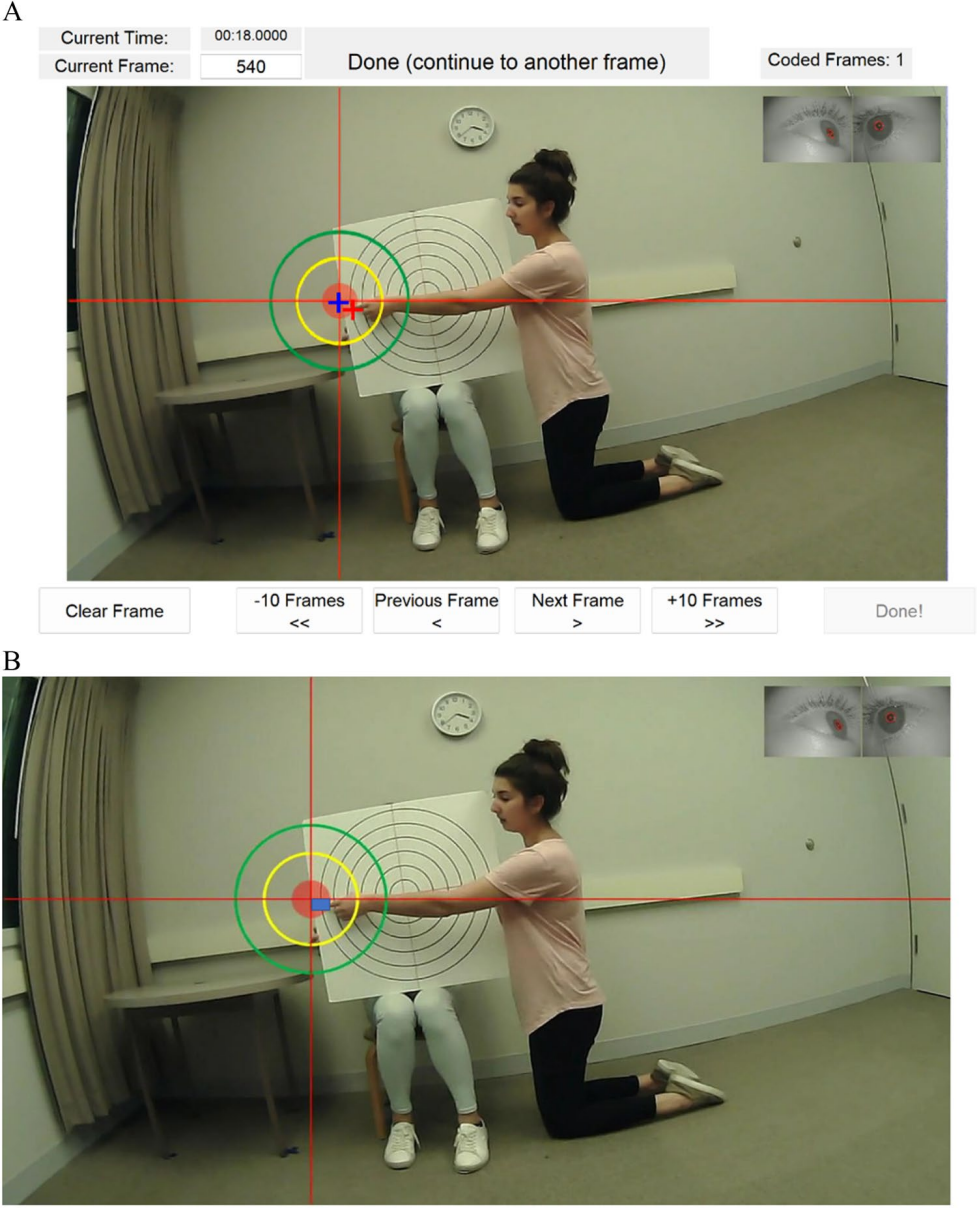
Screenshots of the graphical user interfaces (GUIs) for accuracy calculation. Both the MATLAB tool (**A**) and R Shiny app (**B**) enable users to annotate the point of gaze (i.e., crosshair) and the target location (i.e., where the experimenter is pointing). The data are then used to compute the spatial offset (in degree of visual angle) between the gaze location and the target location. **A** The MATLAB tool takes video inputs. In this example, 25 frames from the validation session were used for the calculation of accuracy. Based on the specifications of Pupil Core eye tracker: fov_x (horizontal field of view in degrees) = 82.1, fov_y (vertical field of view in degrees) = 52.2, fov_res_x (horizontal resolution in pixels) = 1280, fov_res_y (vertical resolution in pixels) = 720. The average spatial offset across these frames is 0.766°. **B** The R Shiny app uses frames extracted from the video recordings. The *blue box* (edited) represents the region between the gaze and the target location. Detailed instructions are provided on https://github.com/JohnFranchak/et_accuracy

We provide an additional tool for calculating the spatial offset (in degree of visual angle) between the gaze location and the validation target that the participant is directed to look at. The tool can be applied to validation recordings obtained from adults or older children in the "MET data collection with adults and older children in controlled laboratory environments" section and calibrated recordings using offline calibration when online calibration is not possible in the "MET data collection in infants and toddlers" and "MET data collection outside controlled laboratory environments" sections. We provide both a MATLAB (https://github.com/xiaoxuefu/MET_methods/tree/main/1.%20Accuracy) and an R Shiny app version of the tool (https://john-franchak.shinyapps.io/Eye-Tracking-Accuracy-Calculator/). The spatial offset computation method is based on the definition described in Franchak and Yu (2022). Figure 1 displays the MATLAB graphical user interface (GUI) and R Shiny app for obtaining the spatial offset. The user can annotate target and gaze locations in the MATLAB GUI or the R Shiny app. Both versions of the tool will compute the spatial offset for each frame based on user-specified target and gaze locations, the scene camera FOV and resolution specifications provided by the manufacturer. A lower spatial offset indicates better accuracy.

Participant-specific accuracy values are recommended for use in scientific reports and making analytical decisions. Participant-specific accuracy is likely to be worse (i.e., larger spatial offset) than manufacturer-reported values (Franchak & Yu, 2022; Niehorster et al., 2020; Santini et al., 2018). Participant-specific accuracy is used to evaluate whether it is valid to determine looking towards AOIs specified for the study. The experimenter should determine the accuracy required to distinguish looking between AOIs. When viewing AOIs at a comparable distance as the validation target, the radius of the AOI, or the distance between two AOIs, should not be smaller than the distance between the target location and the actual point of gaze measured during validation. If the participant-specific accuracy is lower than required, data from the participant may be excluded or the AOI(s) may be adjusted for the participant. For example, the experimenter will determine looking to the person rather than the person’s face for participants with lower accuracy.

### Precision

Precision can also be operationalized as the root mean squared error (RMSE) of sample-to-sample deviation when a participant is assumed to fixate at the same location (Hessels & Hooge, 2019; Niehorster et al., 2020). A larger RMSE value indicates higher sample-to-sample deviation, thus lower precision (Hessels & Hooge, 2019; Niehorster et al., 2020). *GlassesValidator* (Niehorster et al., 2023) provides precision indices using gaze points that are directed at the fixation targets on the poster provided with the tool. We provide a MATLAB program (https://github.com/xiaoxuefu/MET_methods/tree/main/2.%20Precision) for calculating sample-to-sample RMSE based on the published definition (Hessels & Hooge, 2019; Niehorster et al., 2020). The expected input data are *x*- and *y*-coordinates of gaze points when the participants were instructed to look at the same location (i.e., a target object), such as during a calibration procedure. User-input parameters are the scene camera specifications, the size of the target object, and the distance between the participant and the targe object.

Less precise gaze data bias the parcellation of fixations and saccades, as they may erroneously suggest shifts in gaze locations when in fact the gaze remains stable (Wass et al., 2013, 2014). To minimize the impact of low precision, larger AOIs can be defined to allow for more error margins. In addition, data analysis can be less dependent on fixation or saccade categorization by computing the duration of continuous looking toward an AOI (further discussed in the "Gaze annotations").

### Data loss

Data loss can be computed the proportion of data loss (the total amount of valid data points expected to be sampled based on the sampling frequency of the MET device minus the number of valid data points collected) over the total amount of expected data points (Niehorster et al., 2020). Data loss can occur when the eye tracker fails to detect the corneal reflection or pupil (Wass et al., 2014). This can be caused by blinking, lighting, eye camera being moved out of alignment, or other eye tracker technical errors. Hence, with more data loss, shorter durations of AOI looking could be caused by MET failing to detect eye gazes, rather than the participant not looking at the AOI (Wass et al., 2014). Hence, to accurately quantify AOI looking, it is important to measure the amount of both valid and invalid MET data. The amount of AOI looking can then be indexed as the proportion of time looking at the AOI over the total amount of valid MET data recorded (rather than the total recording duration).

## Gaze annotations

### Automated annotations

One challenge in processing MET data is fixation classification. During MET data collection, the participant moves, the AOI moves, or both move in a three-dimensional space. Hence, it is challenging to classify different types of gaze events, including fixations, saccade, and gaze pursuit. For example, during a bout of fixation, the AOI being foveated moves when the participant’s head moves. Classifier algorithms are available for automatic fixation detection (GazeCode: Benjamins et al., 2018; Kothari et al., 2020; Table 1). While the classifiers yielded substantial agreement with human coders, it remains challenging to accurately classify gaze pursuit, defined as the tracking of an AOI moving across the scene camera view (Kothari et al., 2020). However, differentiating fixations from other gaze events or counting the number of fixations might not be the key aims of most MET studies. Depending on the research questions, it might be sufficient to measure the proportion of time, or frames during which, the gaze was directed to an AOI (Franchak & Yu, 2022). This dependent variable can be computed using either manual annotations by human coders or automated classifiers.

Another challenge in MET gaze annotation is identifying the AOI being foveated (Brône et al., 2011. The AOI coordinates need to be defined in the participant-specific egocentric space. They also need to be defined frame-by-frame as the AOI’s appearance can change due to motion, viewing perspectives, and occlusion. Researchers have traditionally conducted manual AOI annotations (e.g., Franchak & Adolph, 2010; Franchak et al., 2011). The development of open-source deep learning algorithms has made it possible to automate AOI identification. Off-the-shelf computer vision algorithms enable automated detection of human faces and bodies in the scene camera view. Once the AOIs (e.g., bounding boxes for faces) are specified, an additional procedure is applied to map the gaze locations (synchronized with the scene camera recordings) to the AOIs (Duchowski et al., 2019; Gehrer et al., 2020; Hessels et al., 2022; Hessels, Benjamins et al., 2020a; Jongerius et al., 2021). Jongerius et al. (2021) found high agreement (Cohen’s kappa ≥ .89) between automated annotation of face looking using *OpenPose* (Cao et al., 2017) and manual annotations by trained coders.

However, computer vision AOI detection can be more challenging for addressing certain research goals than others. A common application has been detecting faces in MET recordings collected during laboratory-controlled face-to-face interactions (Duchowski et al., 2019; Gehrer et al., 2020; Haensel et al., 2022; Hessels, Benjamins et al., 2020a; Jongerius et al., 2021). In contrast, Long et al. (2022) applied *OpenPose* to head-mounted camera recordings obtained from infants during parent–infant free play of toys to detect parents’ wrists as an index of hand presence, as hands are often occluded by the toys. They found more misses in detecting the presence of hands than faces. *OpenPose* detection of human figures is more challenging during unrestrained locomotion when the distance between the wearer and the AOIs varies moment-to-moment (Hessels, Benjamins et al., 2020a). Furthermore, additional training on deep learning models using manually annotated data is required when the AOIs are novel and/or complex objects (e.g., toys; Bambach et al., 2016). Together, automated AOI annotations are faster and can be more objective than manual annotations (Jongerius et al., 2021). The increased data processing compacity can advance our knowledge about the characteristics of visual inputs in the natural environment (Smith & Slone, 2017). However, off-the-shelf computer vision algorithms might not be applicable to all detection tasks. They are also not error-free. Depending on the task requirement and error tolerance, manual annotations might still be necessary for providing training datasets (Bambach et al., 2018) or to complement the automated detection (Haensel et al., 2022).

### Manual annotations

Manual annotations of AOI looking remains the most accessible and robust method for data generation especially for developmental MET applications (Franchak & Yu, 2022). Manual AOI annotations are flexible. As discussed above, it might be a necessary procedure for annotating complex and irregular AOIs. Manual annotations can also be applied to additional events and behaviors that take place simultaneously. Manual annotations are accessible, as it can be carried out in any open-source annotation software, including Datavyu (Datavyu, 2014), ELAN (ELAN, 2018), and BORIS (Friard & Gamba, 2016). Indeed, manual annotations have been widely implemented in studies using a variety of MET systems with both adult (e.g., Laidlaw et al., 2011; Rogers et al., 2018) and child samples (e.g., Franchak et al., 2011; Fu et al., 2019; Woody et al., 2019).

There are two approaches to manual annotations of AOI looking events. One method is manually annotating AOI looking events based on the gaze overlay video entered into annotation software. Researchers may implement a cut-off duration to exclude short looks. For example, an event of continuous looking is conventionally defined as looking at the AOI for two or three successive video frames at 30 Hz, a duration of 66.7 to 99.9 ms (e.g., Franchak & Adolph, 2010; Franchak et al., 2011). The second approach is to apply fixation classifier algorithms to segment the gaze overlay videos into frames based on the detection of stable gazes. Then trained human coders annotate the AOI(s) being looked at in the video segments (e.g., Yurkovic-Harding et al., 2022; Yurkovic et al., 2021). We provide a MATLAB-based *ROI coder* program (https://github.com/JohnFranchak/roi_coder) that aids manual AOI annotations. The computer-program-guided approach can help reduce coders’ cognitive effort and thus reduce human error.

A well-designed gaze annotation manual helps to ease the burden of manual annotations, reduce human errors and biases, and enhance inter-coder reliability. The manual contains descriptions of each code and instructions for the coders on how to score the looking behavior and any additional event of interest. For the looking behavior code, the manual defines the AOI codes (e.g., “b” = body looking) and provides instructions for annotating the onset and offset time of a bout of continuous look to the AOI. Adding to existing guidance (Franchak & Yu, 2022; Slone et al., 2018), we provide best practices for manual annotation of AOI looking events. Best practices for annotating general behavioral data can be accessed at https://datavyu.org/user-guide/best-practices.html.

1. **Create visual aids for manual annotations.** Researchers can superimpose a bullseye on the gaze overlay video to indicate gaze location in addition to the crosshair that presents the point of gaze. The size of the circle can be set based on the tolerance of accuracy for manual annotations. We provide a MATLAB program (https://github.com/xiaoxuefu/MET_methods/tree/main/3.%20Gaze%20Coding%20Error%20Tolerance) for estimating the visual angle of circles in the bullseye (i.e., error tolerance). An example of error margin setting is provided in Fig. 2 (also see Franchak & Yu, 2022 Fig. 4B). Additionally, the gaze overlay video must be synchronized with additional sources of video recordings, such as room cameras. The composite video displays the participant’s behavior from multiple angles and perspectives, thus allowing coders to use contextual information to determine gaze shifts and locations (Slone et al., 2018).

**Fig. 2:**
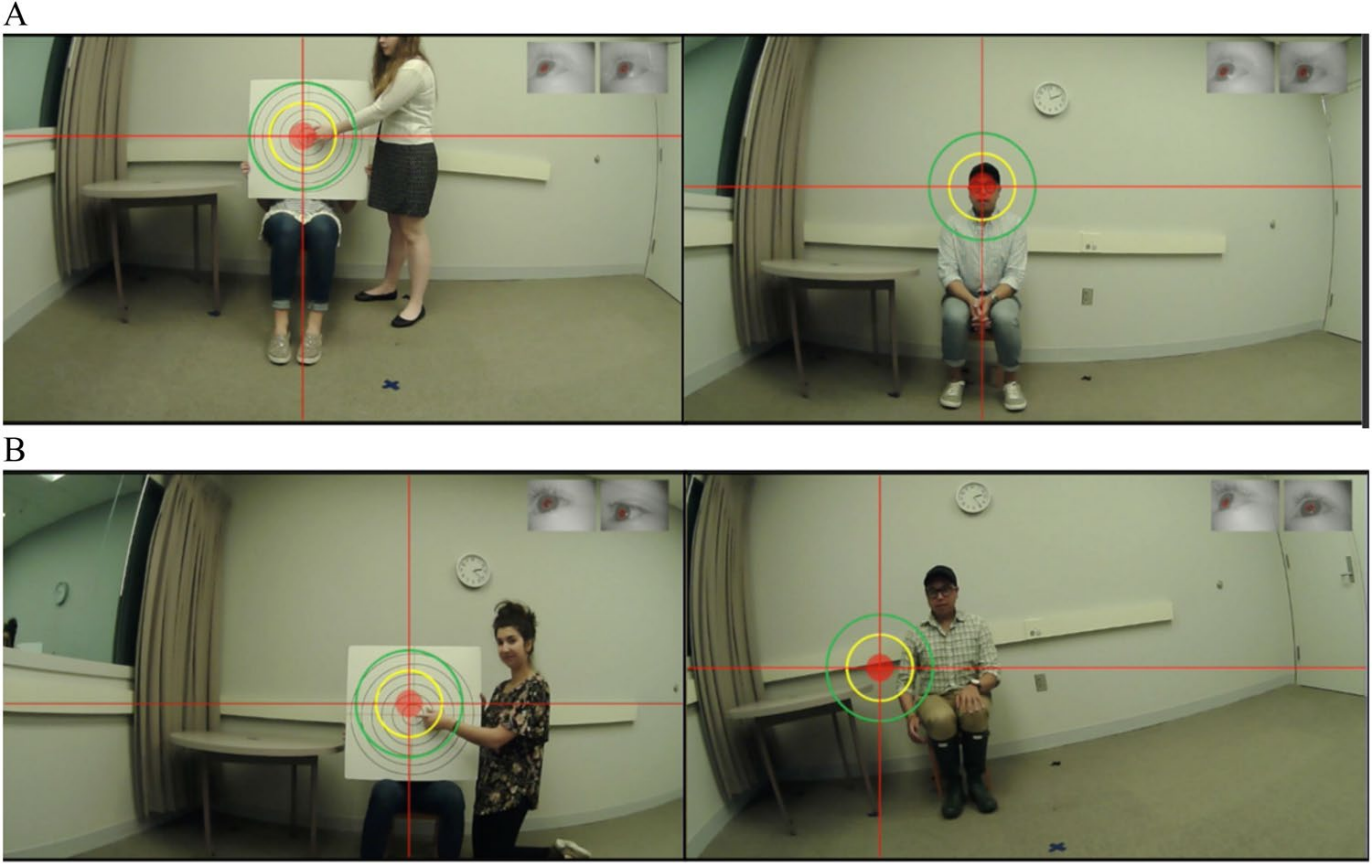
Video frames taken from the validation (*left*) and task (*right*) procedures. Calibration accuracy needs to be estimated before gaze annotations. Gaze annotations that are based on the *red circle* allow reliable determination of the area of interest (AOI) for error within 2.6°. For example, looking to the researcher would be identified for **A** but not **B**. The *yellow circle* allows for an error tolerance of 6.6°. In such case, looking to the researcher would be annotated for **B**
2. **Downsize data to code based on research questions.** Manual annotations can be selective, given the large volume of MET data collected and the time-intensive processing of manual annotations. AOI looking events can be coded only during *events of interest*, instead of the entire recording (e.g., Franchak & Adolph, 2010). Another data-reduction method is to down-sample video frames. The typical sampling frequency of the scene camera is from 30 to 120Hz. For example, a 5-min recording could provide a range of 9000 to 36,000 frames to code. Based on initial inspections of the data, researchers can choose to resample the recordings to a lower frequency if AOIs are relatively big and sparse and gaze shifts within AOIs are not a primary interest of annotations. Researchers can code short segments from several participants at both resampled and the original frequency to make sure that the reduced frame rate does not bias the percentage scores of looking durations.
3. **Annotate valid and invalid AOI looking events and data.** Researchers should compute proportion scores of accumulated AOI looking durations, with the total valid AOI looking duration as the denominator. This strategy is to reduce biases produced by data loss (discussed in the "Data loss" section) and allow for comparisons of accumulated looking durations across AOIs. Thus, in addition to annotating valid AOI looking events (i.e., continuous looking exceeding a threshold duration), coders should annotate frames with invalid AOI looking (e.g., looking duration below the threshold) and data loss (i.e., no visible point of gaze).
4. **Code-check-revise-check.** Manual annotations are an iterative process. After a preliminary annotation plan is conceived, researchers should conduct test annotations of representative recording segments from different participants. This is to make sure that the data generated can address research questions and that satisfactory inter-rater reliability can be easily achieved. Researchers can then go back to revise the annotation methods before annotating the entire recordings. After formal annotation protocol is launched, researchers should periodically check reliability to detect and resolve significant discrepancies between coders. Percentages of inter-coder agreement and kappa values need to be calculated for reliability assessment and scientific reports.

## Data visualization

The gaze annotation step produces a long-format dataset that contains time series of annotated looking events. For example, each row may contain information, such as onset time, offset time, and the AOI. In addition to AOI looking events, Researchers may have coded other events, such as motor activities recorded from the room camera. The multiple types of events, which may be generated from the same individual but different modalities (e.g., looking events and motor behavior), and/or from the same modality but different individuals (e.g., looking events from two individuals during a dyadic interaction). Data visualization is a critical step for exploring the temporal characteristics of AOI looking events from an individual and/or the temporal relations between two or more data streams (e.g., time series of AOI looking and motor behavior). This section will demonstrate the methods and utility of data visualization. The example data and the programs for producing the visualization are shared with the paper.

### Example 1 (iTRAC): Visualize individuals’ looking behavior nested in dyadic interactions

**Fig. 3:**
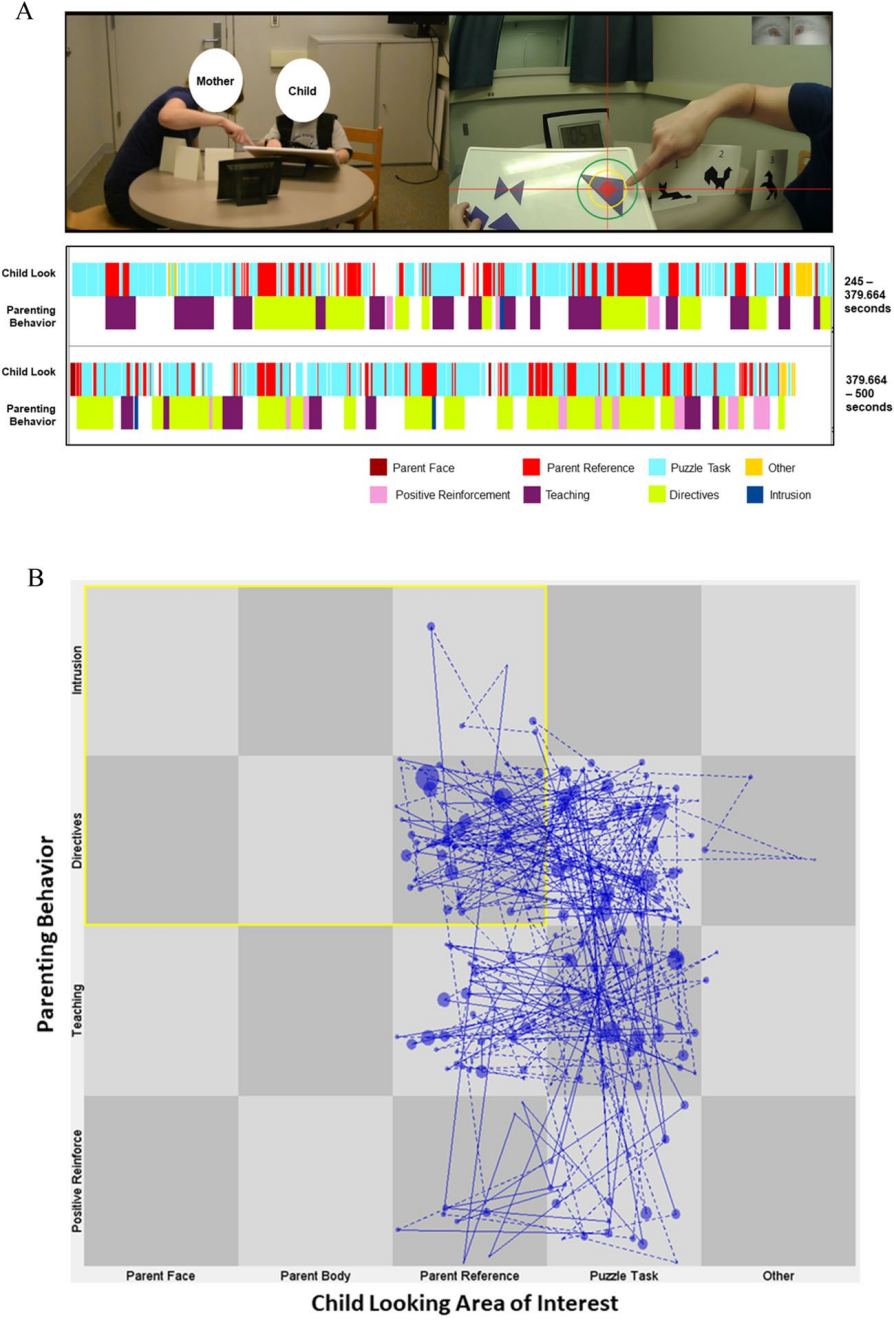
Child–mother dyadic looking behavior nested in parenting behavior. Data are collected from a mother–child dyad as they completed a challenging puzzle task. **A** Screenshot of the composite video used for gaze annotation is displayed in the top panel. The child’s area of interest (AOI) looking events and parent behavior are plotted using the *timevp* MATLAB toolbox. *White gaps* in behavior represent missing child looking behavior (e.g., indeterminate looking or data loss) or parenting behavior (e.g., comforting) that occurred but are not of interest. **B** State space grids (SSGs) depicting the child’s AOI looking event and parent behavior. Each node represents the event when both AOI looking and target parent behavior are co-occurring. The size of the nodes represents the length of time spent in each state. *Lines* between nodes denote changes from one dyadic state to the next. *Dotted lines* connect event nodes prior to missing events to nodes that follow the missing events. The *yellow box* labels the parent-focused/controlling-parenting state

Visualizations inform the temporal dynamics of a child’s looking behavior nested in dyadic interactions. Figure 3 (https://github.com/xiaoxuefu/MET_methods/tree/main/4.%20Visualization/Figure3) presents data collected from a parent–child dyad as they completed a series of challenging tangram puzzles (MacNeill et al., 2022). The visualization explores the child’s gaze patterns as the parent displays various types of parenting behavior (characterized as positive reinforcement, teaching, directives, and intrusion). Figure 3A is plotted using the MATLAB toolbox *timevp* (https://github.com/xiaoxuefu/timevp; Yu et al., 2012) to show how an individual child’s AOI looking events and parenting behavior co-evolve during the task. It shows that there are more teaching behaviors at the beginning of the puzzle task for the dyad. As the time pressure increased as part of the task design, there are more directives and positive reinforcement towards the end of the task. Bouts of looking to the parent become shorter in the second half of the task.

State space grids (SSGs) provide a tool to display how dyadic behaviors vary over time by plotting how members of the dyad move within a figurative space (Hollenstein, 2013; Lewis et al., 1999). Tutorials for using GridWare (https://www.queensu.ca/psychology/adolescent-dynamics-lab/state-space-grids; Lamey et al., 2004) are provided in Hollenstein (2013). Figure 3B demonstrates the utility of SSGs for depicting the temporal dynamics between child looking behavior and parenting behavior. The AOI categories of the child looking events and the types of parenting behaviors form a 5 × 4 grid (i.e., 20 possible dyadic states). We examined the dyad attractor patterns, or states that pull the dyadic system from other states under particular conditions (Thelen & Smith, 1998). GridWare can be used to identify attractors by calculating the average mean duration for a predefined grid sequence, or the average of individual cell means of interest. We characterized attractor strength in parent-focused/controlling parenting states (i.e., the child is looking at the parent while the parent is engaging in directive and intrusive behaviors). In the example, the dyad spent 26.6% of the time, for a total of 37.49 s, in the parent-focused/controlling-parenting states (highlighted in yellow). The average mean duration in these states is 0.85 seconds. Additionally, SSGs help visualize and quantify the patterns of temporal sequence and transition across states (Hollenstein et al., 2004). The level of transition across states, or dyadic flexibility in this example, is indexed by the number of cells visited, the number of transitions, dispersion (0 to 1), and transitional entropy (Lewis et al., 1999), with higher values indicating higher flexibility. The example dyad visited nine cells, made 186 transitions across cells, had a dispersion of 0.83, and an entropy value of 42.22 from looking at the puzzle as the parent engaged in teaching to looking to the parent’s reference as the parent engaged in directive and intrusive behaviors.

### Example 2 (ACTION): Visualize the coordination of multimodal behaviors in triadic parent–infant–object interactions

**Fig. 4:**
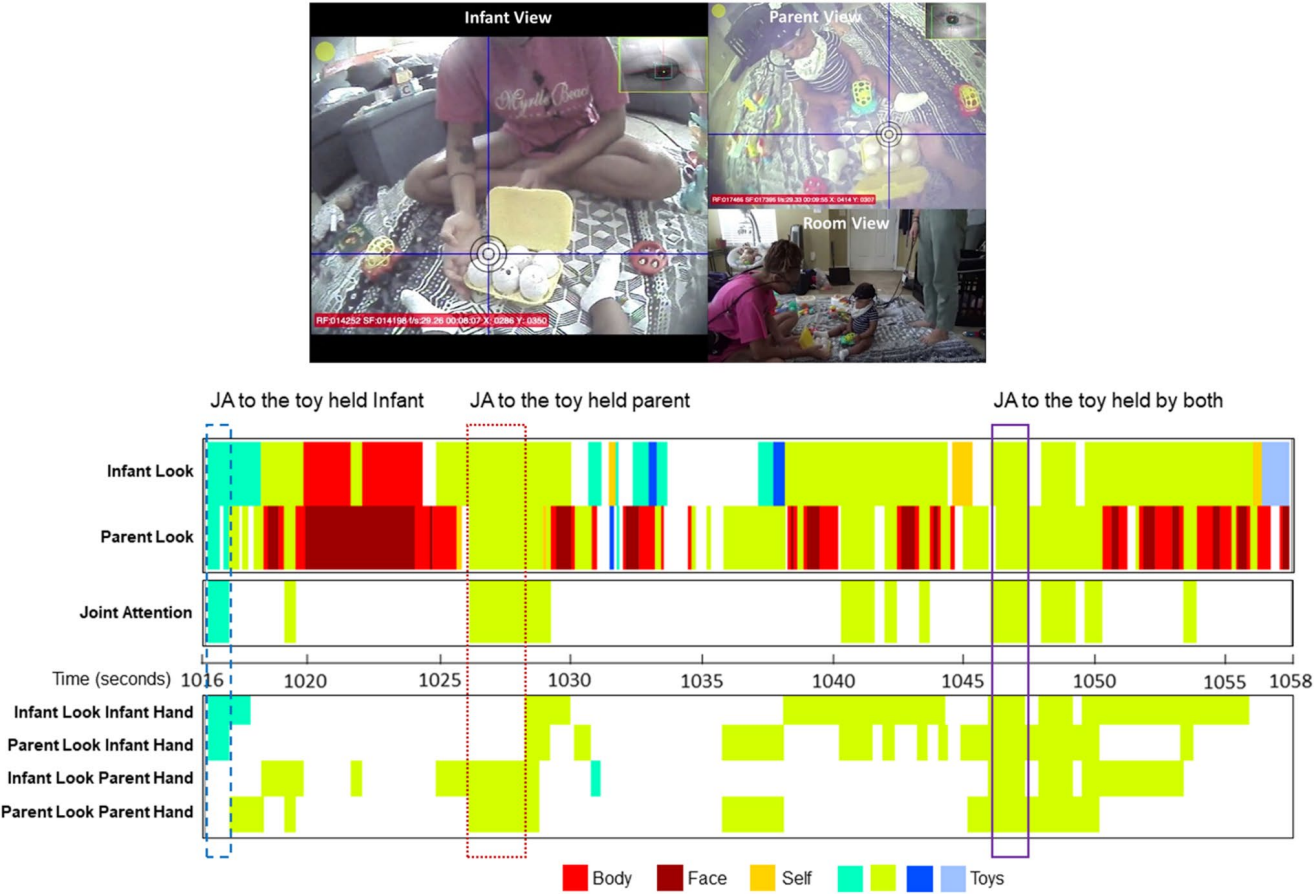
Coordination of looking behavior and manual manipulation of toys between an infant–mother dyad during a segment of free-flowing toy play session. A screenshot of the composite video frame used for gaze annotation is displayed in the top panel. Data were plotted using the *timevp* MATLAB toolbox. The areas of interest (AOIs) for looking behavior are body, face, infant, and mother self-looking and four toy objects. The AOIs for manual manipulation are the toys. *White spaces* denote events that do not involve the AOIs. The first two rows depict raw data from gaze annotation of AOI looking events in the infant and his mother. The third row displays bouts of joint attention (JA), defined as a period (> 0.3 s) when the dyad is looking at the same toy. When the individual is looking at the same toy as the social partner, gaps that last fewer than 0.3 s in consecutive looks of the same AOI are disregarded (e.g., the first bout of toy looking displayed in teal). The last four rows show the four types of attention-manual coordination within each individual and across the dyad

Visualization helps generate higher-order constructs that are defined based on the temporal relations of two or more event types. An example of such construct is joint attention (JA), the ability to coordinate attention with a social partner to an object or event of interest (Tomasello & Farrar, 1986). JA can be measured as the temporal alignment when two individuals are looking at the same object during triadic interactions (i.e., child–parent toy play). Visualizing moment-to-moment temporal relations between looking and bodily behaviors in the dyads over the course of interaction helps (1) identify the occurrence of JA and (2) inform the emergence and impact of JA in real time as the interaction unfolds (Yu & Smith, 2013, 2016, 2017a, 2017b; Yu et al., 2019. Figure 4 (https://github.com/xiaoxuefu/MET_methods/tree/main/4.%20Visualization/Figure4) displays a representative segment of the MET data stream from an 8-month-old. The *timevp* toolbox is used to plot events of interest. The top two rows display raw gaze annotation data of AOI looking events from the infant and his mother. Consistent with existing findings, the infant rarely looked at the social partner (and did not look at the face) during toy play compared to the parent (e.g., Abney et al., 2020; Yu & Smith, 2017a). The third row presents bouts of JA of the toys. For data exploration, we include shorter bouts of JA (between 0.3 and 0.5 s) than Yu and Smith (2017a) considering that the dyad is given a larger variety of toys to play with than the more controlled laboratory setting. The last four rows represent the four combinations of with- and across-individual attention-motor coordination. The three vertical boxes highlight example CVA bouts that emerged when either the partner was holding the toy or both. Consistent with published MET findings (Yu & Smith, 2013, 2017a, 2017b), the figure shows that JA bouts emerge in the context of infant–parent attention-motor coordination.

## Data analysis

### Statistical analysis with aggregated scores

Informed by data visualization, looking event data can be aggregated by AOI and task condition for each participant for subsequent data analysis. Examples of these aggregated, high-order measures include those computed based on the individual’s AOI looking events, such as the number of AOI looks (e.g., Fu et al., 2019; Woody et al., 2019), the temporal characteristics of an individual’s AOI looking events, such as sustained attention, defined as AOI looks that are longer than 3 s (e.g., Yu et al., 2019), and the temporal relations of two individuals’ AOI looking events, such as JA (e.g., Yu et al., 2019). Statistical analysis methods, such as Pearson correlation, linear regression, analysis of variance (ANOVA), and linear mixed effects modeling, can be applied to the aggregated measures. The distribution of the looking behavior measure needs to be carefully inspected so that appropriate data transformation and statistical modeling methods can be selected for non-normally distributed outcome variables.

Several published studies have used analytical strategies using aggregated measures computed based on data visualization (e.g., Abney et al., 2020; Fu et al., 2019; MacNeill et al., 2022; Suarez-Rivera et al., 2019; Woody et al., 2019; Yu & Smith, 2016). For example, using SSGs, MacNeill et al. (2022) examined dyadic states based on co-occurrence of child looking at specific AOIs and designated parenting behavior types (e.g., Fig. 4). Dyadic states were combined to generate two types of attractors: task-focused/positive parenting states and parent-focused/controlling parenting states. Attractor strength, the average mean duration that the child–parent dyad visited each of the two states, is computed. To account for the positive skewness of the attractor strength measures, a generalized linear model with gamma distribution and a log link (Breen, 1996) is fitted to test whether child age, behavioral inhibition, and parent anxiety symptoms predicted the attractor strengths. The results reveal that child age and parent anxiety levels jointly predicted parent-focused/controlling parenting attractor strength.

In another example, Suarez-Rivera et al. (2019) examined the impacts of parent speech and parent manual object manipulation during bouts of JA on infant sustained attention towards the objects during toy play. For each infant, mean proportion scores are computed on infant looking events that feel into five categories defined based on the temporal alignment of multimodal measures: infant looking to the toy without JA, JA with no additional parent behaviors, JA with parent touch of the toy, JA with parent speech, and JA with both parent touch and talk. The resulting mean proportion scores of infant looks are log-transformed to account for the positive skewness. A linear mixed effect model is fitted with the transformed scores as the outcome, event categories as the fixed effect, and random intercepts specified to account for individual differences in the durations of the looking events. The results indicate that infants’ looking to the objects is longest (i.e., greater sustained attention) during bouts of JA that include both parent touch and speech. The aggregated summary scores effectively characterize important behaviors in individuals or dyads. However, analyses with aggregated measures may obscure within-subjects temporal effects that describe how looking behavior changes over time.

### Statistical analysis to model the temporal dynamics of looking events

MET produces a high-density repeated sampling of gaze locations over a prolonged period of data collection, providing a unique opportunity for examining the temporal dynamics of looking behavior. The location and duration of looking behavior change over time within an individual in response to internal and/or external influences. The intensive longitudinal data analysis (Bolger & Laurenceau, 2013) and dynamic systems modeling (Ram & Gerstorf, 2009) approaches provide statistical tools for understanding the patterns and dynamics of intraindividual changes in micro- (e.g., in seconds) and macro-timescales (e.g., in years), investigating factors that modulate the temporal dynamics, and characterize groups of individuals based on the trajectories of changes. The modeling methods have been widely implemented using behavioral observation and self-report data (e.g., Benson et al., 2019; Cole et al., 2020; Morales et al., 2018; Shewark et al., 2020), whereas applications to MET data are limited. However, there is increasing emphasis on a spline-based approach (e.g., Li et al., 2015) to model moment-to-moment nonlinear time-varying effects on AOI looking events (Yamashiro et al., 2019).

**Fig. 5:**
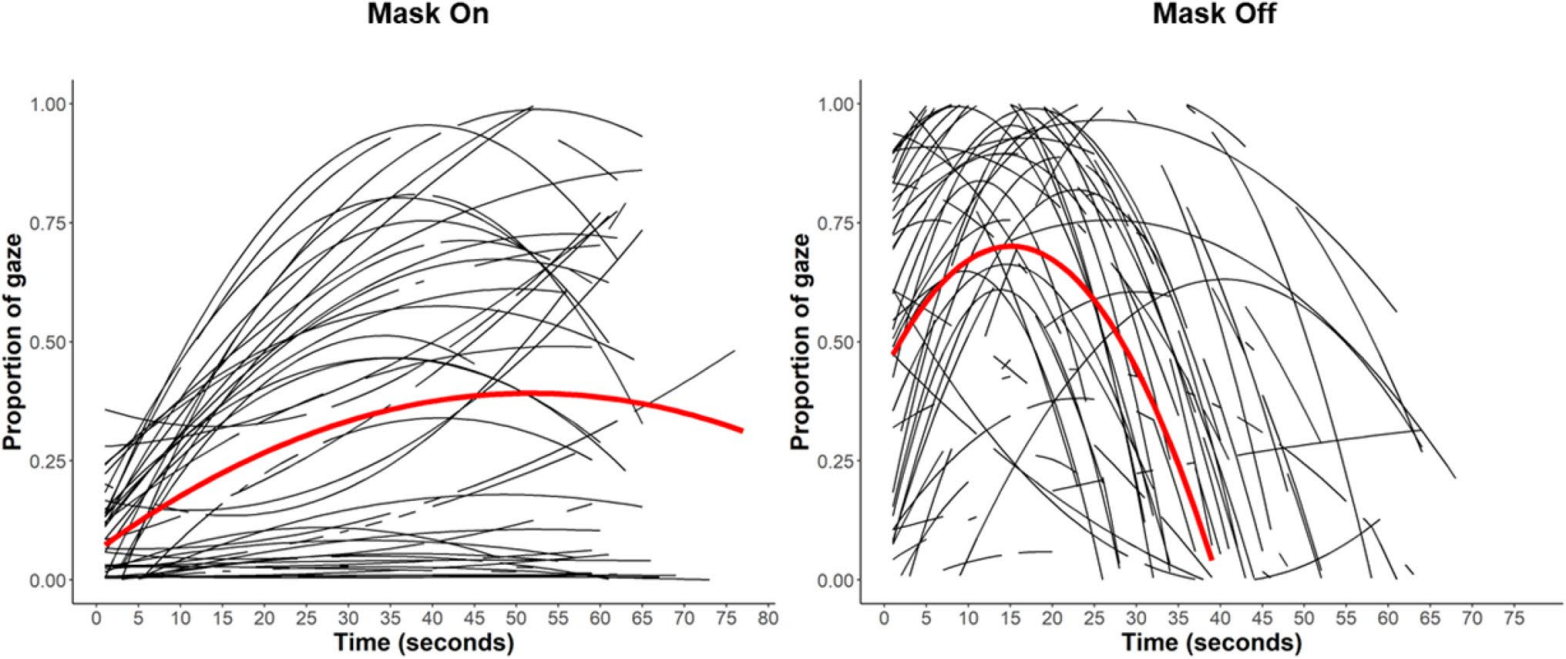
Visualization of the growth curve modeling examining attention to a stranger during the period when the stranger was wearing a scary mask (*left panel*) and when the stranger took off the mask. The visualizations are presented in Figs. 2 and 4 of Gunther et al. (2021). The quadratic trajectory yielded a better fit than a linear trajectory (BIC for a linear fit was 1395.65; BIC for a quadratic fit was 1324.34). The *black lines* show the model-estimated quadratic trajectory for individual participants. The *red line* displays the average quadratic trajectory

Emerging MET studies have modeled interindividual differences in within-subjects temporal trajectories of AOI looking behavior. For example, Gunther et al. (2021) modeled second-by-second changes in looking behavior towards a stranger wearing a gorilla mask in 5- to 7-year-olds. Figure 5 (https://github.com/xiaoxuefu/MET_methods/tree/main/5.%20Data%20Analysis%20-%20Growth%20Model) shows that the looking behavior is characterized by a quadratic trajectory (i.e., inverted U-shape) over the period of exposure. Moreover, Gunther et al. (2021) found a main effect of child behavioral inhibition. As time elapsed while the stranger had the mask on, higher levels of behavioral inhibition were related to a greater proportion of looking toward the stranger. Hence, individual differences in the temperament type shape how looking behavior unfolds over time. Furthermore, in an overlapping sample, Gunther et al. (2022) characterize latent profiles of children based on time-varying trajectories of looking behavior towards a stranger. The stranger pretended to do paperwork without initiating interaction with the child, while also holding the marbles that the child needed to play a game. Similarly, children’s looking behavior exhibits quadratic trajectories over time. Group-based trajectory models (GBTM, Nagin & Odgers, 2010) are fitted to identify latent profiles underlying individual quadratic trajectories. The results indicate that 30.2% of children belong to the “orienting” group, characterized by high initial orienting to the stranger and gradual decay. The rest of the sample is categorized as the “avoidant” group who displays low initial orienting to the stranger and continued low attention. Importantly, individuals’ probability of being characterized with the “avoidant” trajectory predicts variance of internalizing symptoms over and above the aggregated measure of looking towards the stranger. Together, modeling temporal dynamics of looking events may reveal important insights about underlying mechanisms (Cole et al., 2020) and enables better characterization of individual differences (Gunther et al., 2022; Shewark et al., 2020).

## Future directions

We expect to see continuous development in MET hardware that enables MET applications to more diverse samples and data collection environments. Next-generation eye trackers (e.g., Tonsen et al., 2020) are being designed to be calibration-free and more robust to factors that reduce data quality (Niehorster et al., 2020; Valtakari et al., 2021), including participant movement, headset slippage, and changes in ambient lighting. This hardware improvement enables data collection outside the laboratory with participants who have difficulties with online calibration and lower tolerance for the headset. For example, published work has successfully collected MET data for over an hour per session (equivalent to the battery life of the smartphone used for data recording) in toddlers (27- to 31-month-old) as they go about their daily lives at home (Schroer et al., 2022). Future hardware development would benefit from data quality evaluations (e.g., Niehorster et al., 2020) in wider age ranges, clinical populations, and both indoor and outdoor environments.

The increased ease of MET data collection facilitates multimodal research that examines physiological and neural activities concurrently as participants actively attend to external stimuli (Valtakari et al., 2021). An example is to combine MET with functional near-infrared spectroscopy (fNIRS) recording (von Lühmann et al., 2020). fNIRS is a noninvasive neuroimaging tool that measures event-evoked changes in cerebral blood oxygenation. As with electroencephalogram (EEG), fNIRS is well suited for applications in a wide age range (Vanderwert & Nelson, 2014). A key advantage of fNIRS is that robust signals can be obtained even in free-moving participants (e.g., Burgess et al., 2022; Herold et al., 2017). Recent advances in wearable and portable fNIRS devices provide the opportunity to record neural activities in a variety of indoor and outdoor environments as participants actively interact with the environment (Pinti et al., 2020). However, one barrier for the multi-modal data acquisition is signal interference of fNIRS recording, as the eye tracker may emit near-infrared light at a wavelength that can be detected by fNIRS sensors. A specially designed cover for the fNIRS headset is needed to prevent interference (Katus et al., 2019).

MET facilitates research progress in understanding the moment-to-moment unfolding of behavioral and cognitive processes and how those micro-level processes dynamically interact with environmental factors at the macro-level over time. Individuals’ multisensory development, including attention and motor abilities, reciprocally influence the individuals’ social and physical environment throughout the course of human development (Smith et al., 2018). Perturbations in moment-to-moment looking behavior and attention-motor coordination can cast downstream impacts over time and across multiple levels of functioning. MET data collections have been largely implemented in cross-sectional studies, while we know that there are considerable changes in attention and motor functions in the lifespan (e.g., Mason et al., 2019; Reider et al., 2022; Vallesi et al., 2021). Incorporating MET measurements in participants’ naturalistic environment in longitudinal designs can deepen our understanding of how psychological functions that operate in micro-timescales develop with age and give rise to long-term impacts.

## Conclusion

MET allows researchers to sample first-person gaze behavior in the context of ongoing external events, the individual’s behavior, and psychological processes (Hayhoe & Rothkopf, 2011). Commercially available MET hardware allows users to collect good quality data from participants with a wider age range, in various environments, and for longer periods (Franchak & Yu, 2022; Pérez-Edgar et al., 2020). However, challenges in maintaining data quality during acquisition and the lack of standardized protocols for data processing create barriers to applying the technology (Hessels, Niehorster et al., 2020b). This paper provides a practical guide and open-source tools aimed at addressing methodological issues and challenges. This includes maximizing mobility, ensuring MET data quality, good practices in manual gaze annotations, the utility of data visualization, and possible data analytical methods. A number of tools for MET data quality assessment are readily available. This facilitates data quality reporting and data processing. There is increased implementation of automated AOI annotations with the rapid development of computer vision algorithms. However, manual inspections and annotations are indispensable for validating automated AOI annotations and ensure AOI annotation accuracy. Finally, we encourage researchers to utilize the micro-longitudinal structure of MET data to model the temporal dynamics of AOI looking events, in addition to the use of between-subjects aggregated indices (Ram & Gerstorf, 2009). We hope the practical guide can increase the accessibility of MET technology and help to enhance the reliability, standardization, and reproducibility of MET research. In particular, we believe that these methodological advances will propel our conceptual and theoretical understanding of mechanisms that shape behavior, affect, and cognition in-the-moment and cumulatively lay the foundation for long-term or larger-scale patterns of functioning.

## Data Availability

Example data used in data visualizations are available in the GitHub repository: https://github.com/xiaoxuefu/MET_methods.

## Code availability

Programs for data quality inspection, manual gaze annotations, data visualizations and analysis and example datasets are available in the GitHub repository: https://github.com/xiaoxuefu/MET_methods.
